# Cholecalciferol (vitamin D3): efficacy, safety, and implications in public health

**DOI:** 10.3389/fnut.2025.1579957

**Published:** 2025-06-09

**Authors:** Esteban Ortiz-Prado, Jorge Vasconez-Gonzalez, Juan S. Izquierdo-Condoy, Isaac A. Suárez-Sangucho, José Guillermo Prieto-Marín, Karen Bereniss Villarreal-Burbano, Mateo Alejandro Barriga-Collantes, John Alexander Altamirano-Castillo, Domenic Anahi Borja-Mendoza, Jean Carlo Pazmiño-Almeida, María Paz Cadena-Padilla

**Affiliations:** One Health Research Group, Faculty of Health Science, Universidad de Las Americas, Quito, Ecuador

**Keywords:** vitamin D3, cholecalciferol, high doses, effects on health, vitamin D

## Abstract

Vitamin D₃ (cholecalciferol) is a fat-soluble secosteroid with essential roles in calcium-phosphorus metabolism, bone health, and an expanding range of extraskeletal processes. Upon synthesis in the skin via ultraviolet B exposure or ingestion from dietary sources, cholecalciferol is hydroxylated in the liver and kidneys to form its active metabolite, calcitriol (1,25-dihydroxyvitamin D), which exerts pleiotropic effects through vitamin D receptor (VDR)-mediated genomic and non-genomic pathways. This narrative review synthesizes evidence on the systemic effects of high-dose cholecalciferol on bone health, metabolism, cardiovascular and immune function, and its emerging roles in neurological, gastrointestinal, reproductive, oncologic, and psychiatric disorders. High-dose vitamin D₃ has demonstrated benefits in specific populations, including improved bone mineral density, immune homeostasis, glycemic control, and reduced inflammation. In patients with chronic kidney disease, cystic fibrosis, and inflammatory bowel disease, targeted supplementation has been associated with clinical improvements. Preclinical models support calcitriol’s antiproliferative and neuroprotective functions, and its synergistic effects with chemotherapy, although large-scale randomized controlled trials (RCTs) have yielded mixed or inconclusive results, particularly in cancer, cardiovascular events, and cognitive decline. Methodological variability—such as inconsistent dosing regimens, baseline vitamin D status, and heterogeneous populations—limits definitive conclusions. While vitamin D supplementation is generally safe within recommended limits, excessive intake may cause hypercalcemia or nephrolithiasis, emphasizing the need for personalized strategies. Food fortification and targeted screening remain underutilized yet cost-effective public health interventions. Overall, vitamin D₃ represents a promising but complex therapeutic agent, necessitating further rigorously designed clinical trials to establish evidence-based guidelines for its use in diverse pathological conditions.

## 1 Introduction

Vitamin D is a fat-soluble vitamin naturally present in certain foods, fortified in others, and available as a dietary supplement. A distinctive characteristic of vitamin D is its endogenous production in the human body when ultraviolet (UV) rays from sunlight stimulate its synthesis in the skin. The two primary forms of vitamin D are vitamin D2 (ergocalciferol), derived from plants and commonly used in food fortification, and vitamin D3 (cholecalciferol), synthesized in human skin from 7-dehydrocholesterol and obtained from dietary sources of animal origin (1, 2).

**Figure 1:**
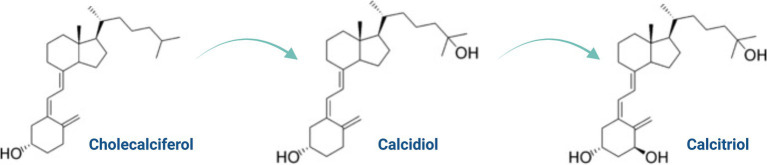
Chemical structure of cholecalciferol, calcifediol, and calcitriol.

The synthesis of vitamin D into its biologically active metabolites occurs through two hydroxylation steps. The first hydroxylation takes place in the liver, where the enzyme 25-hydroxylase converts vitamin D—whether obtained through diet or sunlight—into 25-hydroxyvitamin D. The second hydroxylation occurs in the kidneys, where the enzyme 1-alpha-hydroxylase converts 25-hydroxyvitamin D into the physiologically active form, 1,25-dihydroxyvitamin D (calcitriol) (Figure 1) (3–5). Several tissues, including cardiomyocytes and immune cells, express nuclear vitamin D receptors (VDRs) and respond to 1,25-dihydroxyvitamin D (1,25(OH)2D) (6). While renal hydroxylation dominates systemic calcitriol production, extrarenal activation (e.g., in macrophages) contributes to local immunomodulation (7), serum 25(OH)D cutoffs by ESPEN, 2023 are (e.g., deficiency as <20 ng/mL, insufficiency as 20–30 ng/mL) (8) (Figure 1).

The biological actions of vitamin D are primarily mediated by the VDR, a nuclear receptor expressed in numerous cell types including immune, cardiovascular, and metabolic tissues. Upon binding to calcitriol, the VDR forms a complex with the retinoid X receptor (RXR), translocates to the nucleus, and binds to vitamin D response elements (VDREs) in the promoter regions of target genes. This complex modulates the transcription of genes involved in inflammation, oxidative stress, calcium absorption, and cellular differentiation (9). For example, calcitriol downregulates pro-inflammatory cytokines such as TNF-α and IL-6 by inhibiting NF-κB and MAPK pathways, while enhancing anti-inflammatory cytokines like IL-10 (9, 10). In immune cells, vitamin D enhances the expression of antimicrobial peptides (e.g., cathelicidin, defensins) and promotes regulatory T cell (Treg) differentiation, thus modulating immune tolerance (9, 10).

Vitamin D also regulates genes critical to calcium and phosphate homeostasis, such as *TRPV6* and *calbindin*, which facilitate intestinal calcium absorption (11). Beyond its genomic effects, vitamin D initiates rapid non-genomic signaling cascades involving second messengers and kinases, contributing to processes such as muscle contraction, neurotransmission, and vascular tone (11).

Recent transcriptomic studies have shown that high-dose vitamin D supplementation (e.g., an 80,000 IU bolus) can rapidly alter gene expression profiles in peripheral blood mononuclear cells (PBMCs), upregulating genes involved in focal adhesion (e.g., *HLA-C*) and downregulating pro-inflammatory mediators. However, these responses vary considerably between individuals due to genetic background, baseline vitamin D levels, and epigenetic factors (10). Notably, developmental vitamin D deficiency (DVD) induces persistent epigenetic dysregulation of hepatic genes implicated in cholesterol biosynthesis and energy metabolism, even after postnatal supplementation, suggesting a long-term impact on metabolic disease risk (12).

In metabolic disorders such as type 2 diabetes, vitamin D enhances insulin sensitivity by activating AMPK, suppressing mTOR signaling, and modulating PPAR-γ activity to promote adipogenesis (13). It also regulates microRNAs, such as miR-146a, to attenuate chronic inflammation, a key driver of β-cell dysfunction and insulin resistance (9, 13). These findings underscore vitamin D’s dual role as both a hormonal regulator and an epigenetic modulator linking nutrient status to disease susceptibility. To better contextualize the pleiotropic effects of vitamin D, Table 1 summarizes the key target tissues, downstream pathways, and physiological outcomes mediated by VDR activation across multiple organ systems.

**Table 1:** System-specific effects of vitamin D via VDR activation.

System/organ	Target cells/tissues	Receptor & pathway	Downstream effects	Physiological outcome	Reference
Skeletal System	Osteoblasts, osteoclasts, bone matrix	VDR (Nuclear receptor) → RXR heterodimer → VDRE binding	↑ RANKL expression (osteoclast activation), ↑ osteocalcin, ↑ calcium/phosphate transporters	Bone remodeling, mineralization	(9, 11)
Intestine (GI Tract)	Enterocytes (mainly in duodenum, jejunum)	VDR → ↑ transcription of TRPV6, calbindin-D9k, PMCA1b	↑ Calcium and phosphate absorption	Increases serum Ca^2+^ and Pi	(9, 11)
Kidneys	Distal tubule epithelial cells	VDR → ↑ expression of TRPV5, calbindin-D28k, Na^+^/Pi co-transporters	↑ Calcium reabsorption, ↓ phosphate reabsorption (via FGF23)	Maintains calcium homeostasis, regulates phosphate	(3–5, 11)
Parathyroid Gland	Chief cells	VDR → Direct inhibition of PTH gene transcription	↓ PTH secretion	Negative feedback loop	(9, 11)
Immune System	Monocytes, macrophages, dendritic cells, T cells	VDR → immune gene modulation (e.g., cathelicidin, defensins)	↑ Innate immunity, ↓ Th1/Th17 cytokines, ↑ Treg differentiation	Immunomodulatory: anti-inflammatory & antimicrobial	(9, 10, 13)
Pancreas (β-cells)	Islet β-cells	VDR → Modulates insulin gene expression, Ca^2+^ channels	↑ Insulin secretion (via Ca^2+^ signaling), β-cell survival	Glucose homeostasis	(9, 13)
Cardiovascular System	Vascular smooth muscle cells, cardiomyocytes	VDR → Regulation of renin-angiotensin system, anti-inflammatory genes	↓ Renin expression, ↓ vascular calcification, ↓ proinflammatory cytokines	Cardiovascular protection	(6, 9, 13)
Skin	Keratinocytes	VDR → Regulates proliferation/differentiation	↓ Hyperproliferation, ↑ differentiation (e.g., involucrin, loricrin)	Maintains skin barrier, anti-psoriatic effect	(9)
Reproductive System	Ovarian & testicular cells	VDR → Steroidogenic gene expression	Modulates sex hormone production, folliculogenesis, spermatogenesis	Fertility regulation	(9, 11)
Cancer Cells	Colon, breast, prostate cancer cells	VDR → Cell cycle arrest genes (p21, p27), pro-apoptotic genes	↓ Proliferation, ↑ apoptosis, ↓ angiogenesis	Antitumor effects	(9, 10, 12)

**Figure 2:**
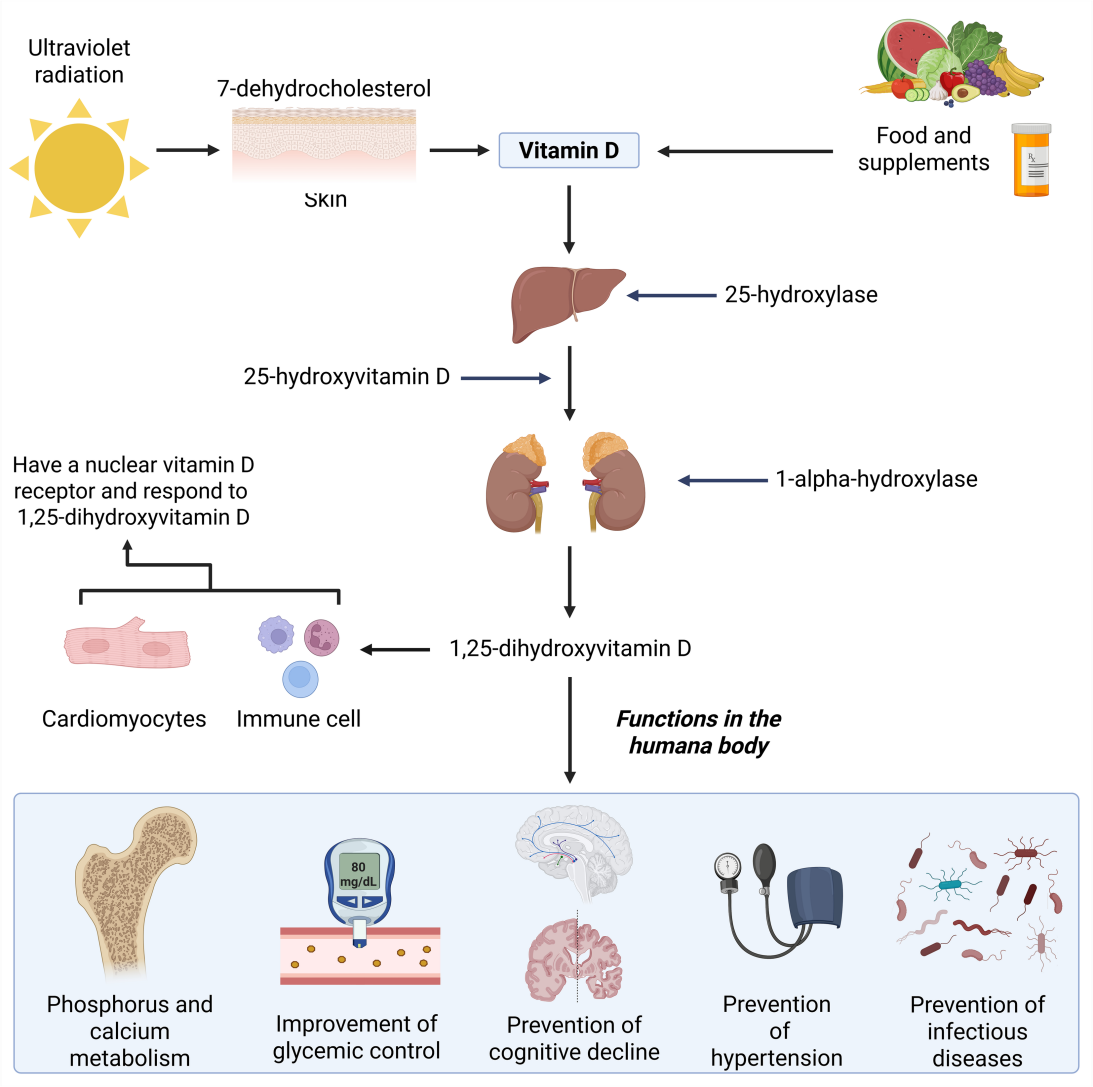
Synthesis and functions of vitamin D in the human body.

Although vitamin D is widely recognized for its role in skeletal health, emerging evidence suggests broader benefits, including improved glycemic control, neurocognitive protection, cardiovascular regulation, and enhanced immunity against infections. Nevertheless, the strength of evidence for these extraskeletal effects remains inconclusive. While observational studies have reported promising associations, randomized controlled trials (RCTs) often yield conflicting results, and optimal dosing strategies are still under debate (14). This duality—established efficacy in bone metabolism versus uncertainty in broader systemic roles—highlights the need for a balanced and evidence-based perspective (Figure 2).

Current parenteral recommendations for vitamin D3 remain conservative at 200 IU/day. Nevertheless, multiple studies have demonstrated that single high-dose regimens—such as 300,000 to 500,000 IU administered once—are both safe and effective, resulting in increases of serum 25(OH)D by approximately 26–28 ng/mL over 1–3 months in elderly or rheumatologic populations (6). Furthermore, maintenance dosing with 50,000 IU weekly or 100,000 IU monthly has been shown to sustain serum 25(OH)D concentrations within the 40–60 ng/mL range, with no evidence of toxicity. These findings reinforce the notion that vitamin D is among the least toxic fat-soluble vitamins, and that vitamin D toxicity is exceedingly rare, especially when serum calcium is monitored (15). This safety profile is partly attributed to the action of CYP24A1, a mitochondrial cytochrome P450 enzyme responsible for the 24-hydroxylation and catabolism of both 25(OH)D and 1,25(OH)₂D. CYP24A1 also metabolizes vitamin D analogs, providing an additional safeguard against excessive accumulation (16, 17).

This narrative review synthesizes current evidence on the systemic effects of high-dose cholecalciferol, focusing on its roles in osteoporosis, diabetes, cardiovascular diseases, gastrointestinal disorders, and cystic fibrosis. It also explores its emerging applications in oncology, neurodegenerative diseases such as Parkinson’s and Alzheimer’s, and other chronic conditions. While numerous reviews have addressed vitamin D in general, this review specifically emphasizes the therapeutic potential and clinical implications of high-dose cholecalciferol across diverse disease contexts.

## 2 Materials and methods

A comprehensive literature review was conducted using Scopus, PubMed, Web of Science, and SciELO databases. No restrictions were applied regarding language or publication date. The following search strategy was employed to identify relevant studies: (“high-dose cholecalciferol” OR “high-dose vitamin D3” OR “cholecalciferol” OR “vitamin D3”) AND (“human health” OR “bone health” OR “fractures” OR “osteoporosis” OR “hypertension” OR “cardiovascular health” OR “atherosclerosis” OR “acute myocardial infarction” OR “atrial fibrillation” OR “inflammatory bowel disease” OR “Crohn’s disease” OR “ulcerative colitis” OR “reproductive health” OR “chronic kidney disease” OR “cystic fibrosis” OR “immune system” OR “neurological health” OR “dermatological disorders” OR “pulmonary diseases” OR “psychiatric disorders”).

All retrieved articles were screened for relevance based on titles and abstracts. Full-text articles that met inclusion criteria were reviewed in detail. In cases where clinical trials were included, their methodological quality was assessed using the CASP Checklist for Randomized Controlled Trials (Supplementary Table S1). For observational studies, including cohort and case–control designs, quality appraisal was performed using the Newcastle-Ottawa Scale (NOS) (Supplementary Tables S2, S3).

## 3 Human health

### 3.1 Bone health

#### 3.1.1 Bone development and maintenance

Vitamin D plays a critical role in bone health by promoting bone formation, mineralization, and maintenance. Supplementation with vitamin D has been shown to enhance collagen fiber production, improve bone material properties, and increase trabecular bone volume fraction. Specifically, higher vitamin D levels contribute to greater bone ductility and hardness, particularly in the tibia. These findings suggest that adequate vitamin D intake during early life may support the attainment of peak bone mass in adulthood, thereby reducing the risk of osteoporosis (7).

High-dose vitamin D3 supplementation (typically 4,000–10,000 IU/day in adults) has been investigated for its potential to promote bone mass accrual and prevent mineral loss. Clinical trials have demonstrated that individuals receiving weekly doses of 20,000 IU of cholecalciferol experience a slower decline in femoral neck bone mineral density (BMD) compared to placebo, suggesting a protective effect against age-related bone loss (18). Mechanistically, vitamin D3 enhances intestinal calcium and phosphorus absorption—two minerals essential for hydroxyapatite deposition and bone matrix mineralization. Inadequate vitamin D3 impairs calcium uptake, weakening skeletal structure and increasing fracture susceptibility. Supporting this, preclinical studies in murine models have shown that high-dose cholecalciferol supplementation results in greater bone mass and mineral content compared to standard-dose regimens (7).

Despite these benefits, high-dose regimens must be interpreted with caution. While doses used in these studies (e.g., 20,000 IU/week) are generally within safe limits for short-term clinical use, the Institute of Medicine (IOM) recommends an upper intake limit of 4,000 IU/day for adults to mitigate the risk of toxicity (19). Chronic excessive intake may lead to hypercalcemia—characterized by elevated serum calcium levels—resulting in clinical manifestations such as nausea, renal dysfunction, nephrocalcinosis, and, in severe cases, vascular calcification or cardiac arrhythmias (19). The risk is especially heightened in individuals with renal impairment, granulomatous diseases, or disorders of calcium metabolism such as hyperparathyroidism.

Beyond its role in bone formation, consistent maintenance of optimal vitamin D3 levels supports lifelong skeletal integrity. Increased serum 25(OH)D concentrations are associated with improved BMD and denser, more resilient bone microarchitecture, particularly during adolescence and early adulthood, when the skeleton is undergoing rapid modeling. This reinforcement of bone structure reduces fracture risk and lays the groundwork for long-term musculoskeletal health. As osteoporosis remains a significant contributor to disability and diminished quality of life among the elderly, maintaining adequate vitamin D status through supplementation represents an effective preventive public health strategy (7).

However, while high doses of vitamin D3 offer clear skeletal benefits, maintaining an appropriate balance is crucial. Excessive vitamin D3 intake can lead to hypercalcemia, characterized by excessive calcium accumulation in soft tissues such as the kidneys and blood vessels, potentially resulting in calcifications and other health complications. Therefore, while vitamin D3 supplementation is a valuable tool for bone growth and maintenance, its administration should be carefully monitored by healthcare professionals. This approach ensures that its benefits are maximized while minimizing potential risks, ultimately supporting long-term skeletal and overall health.

#### 3.1.2 Fracture healing

Vitamin D is substantial for bone maintenance and mineralization by regulating calcium metabolism and maintaining skeletal homeostasis (20). Bone mineralization is essential for fracture healing, particularly in processes such as hard callus formation and bone remodeling (21). Fracture healing occurs in four distinct phases: inflammation, soft callus formation, hard callus formation, and bone remodeling (Table 2).

**Table 2:** Role of vitamin D in the different phases of fracture healing.

Fracture healing phases	Vitamin D role
Inflammation	The alteration of bone and soft tissue continuity results in a fracture hematoma and, consequently, an associated inflammatory response. Vitamin D has been shown to increase the secretion of platelet-derived growth factors. It stimulates the migration and proliferation of mesenchymal stem cells and osteoblasts.
Soft callus formation	Various *in vitro* studies (human bone marrow or osteoblastic cells) have addressed the effect of vitamin D on IGF-I (promotes bone matrix formation), IGF-II (stimulates type I collagen production, cartilage matrix synthesis, and cellular proliferation) and the IGF-binding proteins (3). High doses of vitamin D have been shown to support the production and release of various growth stimulating factors for bone remodeling.
Hard callus formation	Van Driel et al. showed that 1,25(OH)_2_ D_3_ directly stimulated mineralization by activation of vitamin D receptors in the osteoblast, and that the mineralization process was enhanced by the catabolic products of 25(OH)D and 1,25(OH)_2_ D_3_ (24R,25(OH)_2_ D_3_ and 1,24R,25(OH)_3_ D_3_ respectively) (3). Osteoblast proliferation is influenced by vitamin D. In combination with vitamin K, they can modulate the differentiation of human mesenchymal stem cells. Further vitamin D supplementation has been shown to enhance the stimulation of osteoblast genesis. Collagen type I is similarly stimulated by vitamin D supplementation (3).
Bone remodeling	Vitamin D may be an important regulator of osteoclastogenesis, due to the receptors it possesses. There may be a dose-dependent inhibition of osteoclastogenesis by vitamin D, at minimal doses of vitamin D deficiencies there may be inhibition. It has been shown in several studies that vitamin D supplementation significantly increases calcium levels, which contributes to optimal and faster bone remodeling (5).

The potential impact of vitamin D deficiency on fracture healing in humans remains a topic of debate, as most available evidence is derived from indirect and retrospective studies, making causality difficult to establish. However, research conducted on human tissue suggests that vitamin D influences cellular processes involved in fracture healing (22). Despite this, the precise role of vitamin D in human fracture repair has not yet been fully defined. While there is growing interest in the potential benefits of vitamin D supplementation for patients with deficiencies, clinical studies assessing its direct effects on fracture healing remain limited, and no definitive conclusions have been reached (2). A study revealed that vitamin D modulates cellular senescence—a key driver of bone aging—by downregulating p16INK4a and senescence-associated secretory phenotype (SASP) markers in osteoblasts. These pleiotropic effects include enhanced osteogenic differentiation and reduced oxidative stress in bone marrow stromal cells, suggesting a direct anti-aging mechanism (23).

#### 3.1.3 Osteoporosis management

Osteoporosis represents a significant global public health challenge, affecting approximately 25% of the adult population, with postmenopausal women being disproportionately impacted. Characterized by progressive loss of bone mass and microarchitectural deterioration, the condition substantially increases skeletal fragility, rendering patients vulnerable to fractures even after minimal trauma. These fractures can lead to long-term disability, loss of autonomy, and increased mortality, particularly in the elderly.

Management strategies for osteoporosis encompass both pharmacological and non-pharmacological interventions. Pharmacological treatments include bisphosphonates, calcium supplements, and vitamin D, all of which aim to enhance BMD and reduce fracture risk. Among these, vitamin D plays a crucial role by improving calcium absorption, modulating bone remodeling, and supporting muscle function. Clinical studies have shown that vitamin D supplementation, particularly when combined with resistance training or weight-bearing exercise, can significantly improve BMD, reduce fall risk, and enhance overall quality of life in osteoporotic individuals. Additionally, UV light therapy, which promotes endogenous synthesis of vitamin D3, has been proposed as a supportive preventive measure for populations at risk of deficiency.

Although dietary sources such as fatty fish, fish liver oil, and egg yolks contribute to vitamin D intake, they are typically insufficient to maintain optimal serum 25-hydroxyvitamin D [25(OH)D] concentrations in most individuals (24). As such, supplementation is often required, with recommended dosages varying by age and physiological status. For infants up to 1 year, 400–1,000 IU/day is advised; for children and adolescents up to 18 years, 600–1,000 IU/day; and for adults, 1,500–2000 IU/day may be necessary to correct or prevent deficiency. Notably, individuals with obesity may require 2–3 times higher doses due to volumetric dilution and altered metabolism (25).

Current guidelines recommend a combined daily intake of 1,200 mg of calcium and 800 IU of vitamin D to reduce fracture risk in individuals over 65 years and in postmenopausal women (5). Supplementation has been associated with reductions in bone resorption, improved muscle strength and balance, enhanced joint function, and better bone quality—collectively contributing to lower fall and fracture incidence and improved quality of life. Seasonal and geographic factors also influence vitamin D status; for example, in regions with prolonged winter seasons such as Poland, routine vitamin D supplementation is advised.

Despite its benefits, excessive vitamin D intake poses risks, particularly the development of hypercalcemia—a condition marked by elevated serum calcium levels. Symptoms include nausea, vomiting, muscle weakness, polyuria, and in severe cases, renal impairment or nephrolithiasis (26). The risk of toxicity is increased in individuals with comorbidities such as renal insufficiency, sarcoidosis, or hyperparathyroidism.

The relationship between high-dose vitamin D and kidney stone formation remains complex and somewhat controversial. While some studies suggest that excessive vitamin D intake enhances intestinal calcium absorption and urinary calcium excretion—thereby potentially increasing lithogenic risk—others indicate that vitamin D supplementation alone, in the absence of concurrent high calcium intake, does not significantly elevate kidney stone incidence (27). These findings emphasize the need for individualized dosing strategies and close clinical monitoring in high-risk populations.

Importantly, there is no universally accepted definition of what constitutes a “high dose” of vitamin D. Different health authorities propose varying upper intake levels (ULs) based on available safety data. The U.S. National Academy of Medicine recommends a UL of 4,000 IU/day for adults (28), whereas the Endocrine Society suggests that up to 10,000 IU/day may be considered safe for certain individuals under medical supervision, particularly in the context of deficiency or chronic illness (29) (Table 3).

**Table 3:** Comparison of oral and parenteral vitamin D supplementation.

Route of administration	Typical dosing regimens	Bioavailability & pharmacokinetics	Clinical considerations	Reference
Oral (PO)	**Daily:** 600–2,000 IU/day for maintenance; higher doses (e.g., 5,000 IU/day) for deficiency correction. **Weekly:** 50,000 IU/week for 6–8 weeks. **Single high-dose (“Stoss” therapy):** 300,000–600,000 IU once, less commonly used.	Absorption occurs in the small intestine; bioavailability can be affected by factors such as fat malabsorption, gastrointestinal disorders, and concurrent food intake. Peak serum 25(OH)D levels typically achieved within 7–14 days. Requires consistent adherence for sustained levels.	Preferred for general population due to ease of administration and cost-effectiveness. Suitable for maintenance therapy and mild to moderate deficiency. Less effective in individuals with malabsorption syndromes, certain gastrointestinal conditions, or adherence challenges.	(5, 24, 27)
Parenteral (Intramuscular, IM)	**Single dose:** 300,000 IU IM once. **Repeated dosing:** 300,000 IU IM every 3 months, depending on severity of deficiency and patient response.	Bypasses gastrointestinal absorption, leading to more predictable bioavailability. Peak serum 25(OH)D levels may be achieved more rapidly and sustained longer compared to oral dosing. Suitable for patients with malabsorption or non-adherence to oral therapy.	Useful in patients with malabsorption syndromes, severe deficiency, or non-compliance with oral therapy. Requires healthcare professional for administration. Potential for injection site reactions. Monitoring for hypercalcemia is recommended, especially with high-dose regimens.	(26, 27, 29)

Several studies have shown that there is insufficient synthesis of vitamin D through the skin. Deficiencies are more common in patients with osteoporosis and elderly patients. It is necessary to verify the recommended dosage. It is important to perceive vitamin D deficiency to prevent possible fractures or falls (2). The most common are loss of mobility, grip strength and muscle mass.

In postmenopausal women, vitamin D supplementation has been shown to enhance calcium absorption in the intestines. However, routine supplementation is not necessary for women with normal calcium levels. Several studies suggest that dietary supplementation with vitamin D3, in combination with calcium, reduces the risk of falls and fractures, thereby preserving mobility and preventing disability in aging populations (2).

### 3.2 Cardiovascular health

Vitamin D has garnered considerable scientific interest for its potential role in modulating cardiovascular health. Observational studies and meta-analyses have consistently reported associations between low serum 25-hydroxyvitamin D [25(OH)D] concentrations—typically defined as <50 nmol/L—and an increased risk of cardiovascular diseases (CVD) and cardiovascular-related mortality (30). However, findings from large-scale randomized controlled trials (RCTs) remain inconclusive, limiting the ability to draw definitive causal inferences.

One of the most comprehensive investigations to date, the VITAL trial, evaluated the effects of daily supplementation with 2,000 IU of vitamin D3 and found no statistically significant reduction in the incidence of major cardiovascular events in the general population. Nonetheless, subgroup analyses suggested potential cardiovascular benefits in specific cohorts, such as individuals with low baseline vitamin D levels or those with limited sun exposure (21). Similarly, the D-Health trial yielded mixed results, underscoring the complexity and heterogeneity of vitamin D’s cardiovascular effects (31). These discrepancies may be attributed to differences in baseline 25(OH)D status, supplementation regimens, study duration, or population characteristics, as well as the potential for threshold effects in vitamin D response.

At the mechanistic level, vitamin D exerts its biological effects through the vitamin D receptor (VDR), which is expressed in various cardiovascular cell types, including endothelial cells, cardiomyocytes, and vascular smooth muscle cells (32). VDR activation influences numerous pathways relevant to cardiovascular function, such as inhibition of the renin-angiotensin-aldosterone system (RAAS), modulation of inflammatory cytokines, and regulation of vascular tone and calcification. These findings suggest that vitamin D may play a direct role in maintaining cardiovascular homeostasis.

Meta-analyses of observational studies have reported a U-shaped relationship between vitamin D status and CVD risk, with optimal serum 25(OH)D levels around 75 nmol/L being associated with the lowest cardiovascular risk (33). However, this association has not been consistently replicated in interventional trials, calling into question the causality of the relationship. Additionally, Mendelian randomization studies—designed to assess causality using genetic variants—have largely failed to demonstrate a clear benefit of genetically determined higher vitamin D levels on cardiovascular outcomes (34).

Standard-dose vitamin D supplementation (2,000–4,000 IU/day) is generally considered safe and well-tolerated, with minimal risk of adverse effects. However, excessive intake (>10,000 IU/day) may lead to hypercalcemia, a rare but serious complication that can result in arrhythmias, vascular calcification, or electrocardiographic changes mimicking acute myocardial infarction (30). Therefore, supplementation strategies should be guided by baseline serum levels, patient-specific risk factors, and current clinical guidelines.

#### 3.2.1 Hypertension

Hypertension is a major cardiovascular risk factor with a multifactorial etiology in which vitamin D may play a significant role. Large-scale studies, such as NHANES, have reported an inverse relationship between serum 25(OH)D (25-hydroxyvitamin D) levels and blood pressure, particularly in individuals with moderate to severe deficiency. This effect may be mediated through vitamin D’s ability to suppress the renin-angiotensin-aldosterone system (RAAS), enhance endothelial nitric oxide synthase (eNOS) activity, and reduce cyclooxygenase-1 (COX-1) activity, thereby promoting vasodilation and reducing arterial stiffness (35). Additionally, vitamin D has been associated with an improved lipid profile, including reduced triglycerides and total cholesterol, along with increased HDL-C levels (32).

Experimental studies in VDR-knockout mice have demonstrated cardiac hypertrophy and elevated blood pressure due to RAAS activation. However, studies using calcium-, phosphate-, and lactose-corrected diets in the same models suggest that vitamin D’s vascular effects may be independent of RAAS modulation. Furthermore, some studies indicate that vitamin D supplementation primarily benefits individuals with comorbidities such as diabetes or cardiometabolic disease (35).

There is also evidence indicating a reduction in systolic blood pressure (SBP) in patients with Type 1 Diabetes Mellitus (T1DM) and cardiovascular autonomic neuropathy (CAN) following high-dose cholecalciferol supplementation (33). Nevertheless, other studies and meta-analyses have reported that supplementation improves endothelial function and reduces blood pressure, although not all findings have been consistent (32).

#### 3.2.2 Atherosclerosis and heart failure

Vitamin D plays a role in mitigating oxidative stress by activating antioxidant enzymes such as glutathione peroxidase and superoxide dismutase, thereby improving nitric oxide (NO) bioavailability, which is essential for vasodilation and reducing inflammation (32). Given its influence on plaque formation and vascular inflammation, vitamin D is hypothesized to impact the progression of atherosclerosis. However, clinical evidence is conflicting, with some studies reporting improvements in inflammatory markers while others fail to demonstrate significant effects (36).

Large-scale trials such as VITAL and ViDA have not shown a clear benefit of vitamin D supplementation in improving cardiac function, preventing fibrosis, or reducing left ventricular hypertrophy in individuals with adequate vitamin D levels (36). However, severe vitamin D deficiencies, such as those seen in rickets and osteomalacia, have been linked to heart failure, conditions that can be reversed with appropriate vitamin D and calcium supplementation (33).

#### 3.2.3 Acute myocardial infarction (AMI)

The Framingham study found that individuals with vitamin D deficiency have an 80% higher risk of developing acute myocardial infarction (AMI). This is believed to be mediated through vitamin D’s regulation of the RAAS, specifically through the modulation of the angiotensin II receptor (ACE2) and the reduction of angiotensin II levels, which yield cardioprotective effects (37). Additionally, vitamin D deficiency has been linked to poorer left ventricular function, increased risk of sudden cardiac death, and elevated expression of genes associated with inflammation and fibrosis, suggesting a potential protective role for vitamin D in cardiovascular health (32).

However, further research is needed to determine whether vitamin D supplementation can significantly reduce AMI risk (37). Studies conducted in Denmark since 1978 and more recent investigations, such as those by Ng et al., have shown that 74% of AMI patients had low vitamin D levels, which were associated with an increased risk of in-hospital mortality and long-term adverse outcomes (37).

#### 3.2.4 Atrial fibrillation (AF)

Atrial fibrillation (AF) is the most common arrhythmia, associated with an elevated risk of stroke, increased mortality, and a significant healthcare burden. While observational studies suggest that individuals with vitamin D deficiency have nearly twice the risk of developing AF, potentially due to mechanisms involving RAAS activation, atrial remodeling, and inflammation, findings from prospective studies remain inconsistent (37). In the context of postoperative AF, particularly after coronary artery bypass grafting, low vitamin D levels have been associated with an increased risk of this condition. A recent meta-analysis reported a 12% higher incidence of postoperative AF in patients with vitamin D deficiency. Conversely, supplementation in patients with severe deficiency (<20 ng/mL) has been linked to a modest reduction in postoperative AF risk, suggesting that vitamin D may have potential benefits in specific clinical scenarios (37) (Table 4).

**Table 4:** Summary of vitamin D effects on cardiovascular health.

Cardiovascular aspect	Key findings	Mechanisms	Research gaps	Reference
General Cardiovascular Health	Low vitamin D (<50 nmol/L) is associated with increased CVD risk in observational studies	VDRs in endothelial cells, cardiomyocytes, and vascular smooth muscle cells; influences inflammation and vascular tone	Need for RCTs in deficient populations; unclear optimal dose	(21, 30)
Hypertension	Inverse correlation between 25(OH)D levels and blood pressure, especially in deficient individuals	RAAS suppression, ↑eNOS activity, ↓COX-1, improved lipid profile	Effectiveness in normotensive or mildly hypertensive individuals remains unclear	(32, 33, 35)
Atherosclerosis & Heart Failure	Suggested role in reducing oxidative stress and vascular inflammation; mixed clinical results	↑Antioxidant enzymes (e.g., SOD, GPx), ↑NO bioavailability	Conflicting data; effect may be limited to severe deficiency	(32, 33, 35, 36)
Acute Myocardial Infarction (AMI)	Vitamin D deficiency linked to ↑AMI risk and worse outcomes	↓Angiotensin II, modulation of ACE2, anti-inflammatory effects	Uncertain if supplementation lowers AMI incidence or improves prognosis	(32), (37)
Atrial Fibrillation (AF)	Deficiency associated with ~2 × AF risk; 12% higher postoperative AF in deficient patients	RAAS activation, atrial remodeling, inflammation	More studies needed in surgical and high-risk populations	(37)

### 3.3 Gastrointestinal health

#### 3.3.1 Inflammatory bowel disease

Vitamin D plays a key role in inflammatory bowel disease (IBD), which encompasses conditions such as Crohn’s disease and ulcerative colitis. Beyond its well-established functions in calcium and phosphorus homeostasis, vitamin D exerts immunomodulatory effects that may influence IBD pathogenesis. Epidemiological studies have demonstrated an association between vitamin D deficiency and an increased risk of developing IBD, as well as poorer clinical outcomes, including higher hospitalization rates and an increased need for surgical interventions. Additionally, IBD itself contributes to vitamin D deficiency due to malabsorption, dietary restrictions, and reduced sun exposure. This bidirectional relationship suggests that maintaining optimal vitamin D levels may be critical for effective disease management (38).

Clinical evidence suggests that vitamin D status may influence disease activity and therapeutic efficacy. In a retrospective study of 88 patients with IBD receiving vedolizumab, higher baseline serum 25(OH)D concentrations (≥30 ng/mL) were associated with improved endoscopic response in UC and elevated drug levels in CD, suggesting a potential synergistic effect between vitamin D and biologic therapy (39). Furthermore, a systematic review of nine clinical trials found that daily supplementation with ≥2,000 IU of vitamin D significantly improved clinical disease indices and quality of life in patients with active IBD, although considerable heterogeneity across studies precludes definitive recommendations on optimal dosing (40).

In pediatric populations, the relationship between vitamin D status and disease activity has also been observed. A study involving 96 children with IBD showed that serum 25(OH)D levels were significantly lower during active disease phases compared to periods of remission. Moreover, vitamin D deficiency exhibited notable seasonal variation, highlighting the importance of monitoring and timely supplementation, particularly during winter months or periods of increased disease activity (41).

Emerging evidence supports the use of high-dose vitamin D supplementation (typically 4,000–10,000 IU/day) to modulate intestinal inflammation, improve epithelial barrier integrity, and favorably alter the composition of the gut microbiota. Targeting serum 25(OH)D concentrations between 40 and 60 ng/mL may confer therapeutic benefits in terms of reducing disease flares and maintaining remission (38). However, further randomized controlled trials are needed to establish evidence-based guidelines for dosing, duration, and monitoring of vitamin D supplementation in both adult and pediatric IBD populations.

#### 3.3.2 Crohn’s disease

Vitamin D plays a fundamental role in immune regulation and intestinal homeostasis. Deficiency is particularly prevalent in patients with Crohn’s disease, with some clinical studies reporting rates as high as 100% in certain populations (42). This deficiency is primarily attributed to intestinal malabsorption, dietary restrictions, and reduced sun exposure, further exacerbated by the chronic inflammation characteristic of the disease.

In its active form (1,25-dihydroxyvitamin D), vitamin D modulates the immune response by regulating T-cell activity, promoting the production of anti-inflammatory cytokines such as IL-10, and suppressing proinflammatory mediators like TNF-α. Preliminary studies suggest that vitamin D supplementation may enhance intestinal barrier integrity, reduce mucosal inflammation, and contribute to sustained clinical remission (42).

For patients with severe vitamin D deficiency, recommended dosing includes 6,000–10,000 IU/day or weekly loading doses of 50,000 IU for 8–12 weeks. These regimens have been shown to decrease inflammation, regulate proinflammatory T-cell subsets (Th1 and Th17), and strengthen the intestinal epithelial barrier, highlighting the potential of vitamin D as an adjunctive therapy in Crohn’s disease management (42).

#### 3.3.3 Ulcerative colitis

In ulcerative colitis, vitamin D exerts a critical immunomodulatory effect through its interaction with the nuclear VDR, which regulates genes involved in inflammation and tissue repair. VDR activation has been shown to suppress NLRP6 inflammasome activity, reducing the production of inflammatory cytokines such as interleukin-1β (IL-1β) and interleukin-18 (IL-18). These effects help limit structural damage to the intestinal epithelium and maintain mucosal homeostasis. Experimental models in both animals and human studies suggest that vitamin D3 supplementation not only reduces acute inflammatory responses but also lowers relapse rates, reinforcing its potential as a therapeutic agent in ulcerative colitis (43).

Vitamin D_3_ at 4,000 IU/day is more effective than 2,000 IU/day in increasing vitamin D to sufficient levels in UC patients with hypovitaminosis D (44). These doses have been associated with enhanced colonic epithelial protection, immune system regulation, and improved intestinal barrier function. However, supplementation should be medically supervised to prevent toxicity and ensure therapeutic efficacy (43) (Table 5).

**Table 5:** Summary of vitamin D effects on gastrointestinal health.

Condition	Key findings	Mechanisms	Supplementation evidence	Research gaps	Reference
Inflammatory Bowel Disease (IBD)	Deficiency linked to higher IBD risk, worse outcomes, and need for surgery	Immunomodulation, improved barrier function, anti-inflammatory effects	≥2,000 IU/day improves clinical scores; ≥30 ng/mL serum 25(OH)D improves biologic drug response (e.g., vedolizumab)	Optimal dosing strategies; need for large RCTs	(38, 39, 41)
Crohn’s Disease	Up to 100% deficiency rates in some populations; affects remission and inflammation control	↓TNF-α, ↑IL-10, T-cell regulation, improved mucosal integrity	High-dose vitamin D reduces inflammation and supports remission	Limited data on long-term safety and efficacy	(42)
Ulcerative Colitis	VDR activation reduces inflammasome (NLRP6), cytokines (IL-1β, IL-18); lowers relapse risk	VDR-mediated suppression of epithelial damage and inflammation	Vitamin D3 reduces relapse rates and enhances barrier protection	More trials needed to confirm clinical endpoints and relapse prevention	(43, 44)

### 3.4 Glucose metabolism and diabetes

The relationship between vitamin D and glucose metabolism has garnered growing attention due to the rising global burden of diabetes and metabolic syndrome. Vitamin D receptors (VDRs) are expressed in pancreatic β-cells, adipocytes, and skeletal muscle—tissues involved in glucose homeostasis—suggesting a potential regulatory role in insulin secretion and sensitivity.

One large-scale randomized clinical trial involving 1,774 participants assessed the impact of daily supplementation with 4,000 IU of vitamin D3 over a 24-month period. The study found no significant improvement in β-cell function as measured by oral glucose tolerance test (OGTT)-derived indices in individuals with prediabetes who were not selected based on baseline vitamin D status (45).

Nevertheless, multiple meta-analyses suggest that vitamin D supplementation may confer metabolic benefits, particularly in vitamin D-deficient individuals or those with prediabetes. A meta-analysis of 28 randomized controlled trials, comprising 3,848 participants, demonstrated that vitamin D supplementation significantly reduced glycated hemoglobin (HbA1c), fasting plasma glucose, and HOMA-IR—a validated surrogate marker of insulin resistance. These improvements were most pronounced in subgroups receiving co-supplementation with calcium, as well as in women, individuals over 50 years of age, and those with gestational diabetes mellitus (46).

Mechanistically, vitamin D deficiency is inversely correlated with insulin sensitivity. Low serum 25(OH)D levels have been associated with higher HOMA-IR scores, indicating increased insulin resistance. Vitamin D may exert its insulin-sensitizing effects through multiple pathways, including enhanced expression of insulin receptors, modulation of inflammatory cytokines (e.g., TNF-α, IL-6), and regulation of VDR polymorphisms within pancreatic β-cells. Additionally, vitamin D facilitates the conversion of proinsulin to insulin, improves insulin receptor phosphorylation, and augments glucose transporter expression, thereby promoting glucose uptake in peripheral tissues (32).

Furthermore, vitamin D influences both genomic and non-genomic signaling mechanisms. Genomically, it acts through VDRE-mediated transcription in liver, muscle, and adipose tissue. Non-genomically, vitamin D activates second messenger pathways that acutely enhance insulin signaling. In individuals with elevated body mass index (BMI), vitamin D deficiency has been linked to increased systemic inflammation, characterized by elevated proinflammatory cytokines that contribute to insulin resistance (45).

Short-term intervention studies suggest that vitamin D supplementation may reduce glycated hemoglobin (HbA1c) levels, potentially delaying the onset and progression of diabetes-related complications (47). Moreover, studies have reported a significant reduction in HOMA-IR following vitamin D supplementation, highlighting its potential role in improving insulin sensitivity. Vitamin D deficiency has also been linked to diabetic nephropathy and both microvascular and macrovascular complications in diabetes (48).

While the exact mechanisms by which vitamin D regulates glucose metabolism remain unclear, current evidence suggests that it plays a crucial role in insulin production and sensitivity. VDRs in pancreatic beta cells are essential for insulin secretion, and vitamin D supplementation has been shown to improve beta cell function and insulin sensitivity, particularly in individuals with low baseline vitamin D levels. However, the effectiveness of vitamin D supplementation varies depending on the method used to assess outcomes.

Despite inconsistent results in clinical trials, meta-analyses suggest that vitamin D supplementation may help reduce the risk of diabetes progression and improve glycemic control. Additionally, maintaining adequate vitamin D levels may contribute to type 1 diabetes prevention and reduce complications associated with type 2 diabetes and gestational diabetes. Given the potential metabolic benefits of vitamin D, further well-designed randomized controlled trials are needed to clarify its role in diabetes prevention and management.

### 3.5 Lipid metabolism

Research has demonstrated that the production, storage, and metabolism of the active form of vitamin D occur within adipose tissue. Adipocytes (fat cells) express both the vitamin D receptor (VDR) and the membrane-associated rapid response steroid-binding protein (1,25D-MARRS), allowing vitamin D to exert genomic effects (which influence gene expression) and non-genomic effects (which mediate rapid cellular responses) in adipose tissue. These effects regulate key processes such as adipogenesis, apoptosis, inflammation, and adipokine secretion (49).

Vitamin D plays a critical role in the formation and function of adipocytes, influencing their development, activity, and metabolic properties. However, despite growing evidence supporting its role in adipose tissue regulation, further clinical research is needed to fully elucidate the impact of vitamin D supplementation in individuals with metabolic disorders and obesity (49).

Changes in adipocyte quantity and size significantly affect the surrounding adipose tissue microenvironment. These alterations influence adipokine secretion, fatty acid metabolism, and hypoxia, and can lead to adipocyte apoptosis, impaired fatty acid transport, and chronic low-grade inflammation. Adipose tissue also contains immune cells that participate in adaptive immune responses, such as the clearance of apoptotic fat cells, differentiation of new adipocytes, and angiogenesis. In obesity, these regulatory mechanisms become dysregulated, contributing to persistent inflammation and metabolic disorders, including insulin resistance (49).

Insulin is a key regulator of triglyceride (TG) accumulation in adipocytes. It promotes glucose uptake and lipogenesis, stimulates pre-adipocyte differentiation into mature adipocytes, and inhibits lipolysis. Vitamin D influences adipocyte apoptosis through mechanisms closely linked to calcium homeostasis, which it helps regulate (50).

Studies have shown that vitamin D’s effects on lipolysis and lipogenesis vary depending on the type of adipose tissue. Specifically, vitamin D stimulates lipolysis in visceral adipose tissue but decreases this process in subcutaneous tissue. Conversely, vitamin D promotes lipogenesis in subcutaneous tissue while inhibiting it in visceral adipose tissue (50).

Vitamin D plays a multifaceted role in adipose tissue by influencing both the formation and function of adipocytes through genomic and non-genomic mechanisms. Its effects on lipid metabolism vary between visceral and subcutaneous fat, highlighting its intricate regulatory functions. Despite promising insights into vitamin D’s impact on lipolysis, lipogenesis, and adipose tissue inflammation, further clinical research is necessary to fully understand its therapeutic potential in metabolic disorders and obesity.

### 3.6 Vitamin D importance in reproductive health and pregnant women

#### 3.6.1 Reproductive health

Vitamin D, primarily through its active metabolite calcitriol, regulates the transcription of genes involved in steroidogenesis—such as CYP19A1 (aromatase), StAR (steroidogenic acute regulatory protein), and 3β-HSD (3β-hydroxysteroid dehydrogenase)—as well as genes controlling follicular development, endometrial decidualization, and semen quality via VDR-mediated genomic signaling pathways (51, 52). By enhancing the expression of estrogen- and progesterone-synthesizing enzymes in granulosa and theca cells, vitamin D supports ovarian follicle maturation and improves oocyte quality. Its immunomodulatory effects on the endometrium promote vascular remodeling and facilitate embryo implantation (53, 54).

In males, VDR activation contributes to androgen biosynthesis and improves sperm motility. Vitamin D deficiency has been associated with oligozoospermia and reduced semen quality (55). Notably, maintaining serum 25(OH)D levels ≥50 ng/mL has been correlated with higher clinical pregnancy and live birth rates in both *in vitro* fertilization (IVF) and natural conception settings, highlighting vitamin D status as a modifiable factor influencing fertility and reproductive outcomes (56).

#### 3.6.2 Vitamin D supplementation and gestational diabetes (GD)

Vitamin D insufficiency and deficiency during pregnancy have been associated with increased risks of hyperglycemia, insulin resistance, and the development of type 2 diabetes mellitus (T2DM) and gestational diabetes (GD). These associations are attributed to vitamin D’s role in promoting insulin secretion from pancreatic β-cells and enhancing insulin receptor expression, both essential for maintaining glucose homeostasis (57). When vitamin D levels are suboptimal, these mechanisms are impaired, predisposing pregnant women to metabolic disturbances that can adversely affect both maternal and fetal health.

Meta-analyses of observational studies have reported significant associations between low maternal serum 25-hydroxyvitamin D [25(OH)D] levels and a higher risk of gestational diabetes. One meta-analysis involving 20 studies and more than 9,000 participants found that vitamin D deficiency was associated with a 53% increased risk of GD (odds ratio [OR] = 1.53; 95% confidence interval [CI]: 1.33–1.75) (58).

Vitamin D3 deficiency in pregnancy has also been linked to a higher incidence of preeclampsia, preterm birth, small-for-gestational-age (SGA) infants, and impaired neurodevelopment in offspring, suggesting that its impact extends beyond glucose metabolism and affects broader aspects of maternal and neonatal health (59). Regarding GD specifically, a case–control study involving 50 participants did not find a statistically significant association between vitamin D3 deficiency and GD incidence. However, it did observe that individuals with both GD and low vitamin D3 levels had values within the “insufficient” range, supporting the hypothesis that vitamin D may influence insulin regulation and contribute to gestational glycemic outcomes (60). Despite these findings, further well-designed, adequately powered RCTs are needed to clarify the causal relationship between maternal vitamin D status and gestational diabetes risk.

#### 3.6.3 Vitamin D supplementation and pre-eclampsia

Preeclampsia is a hypertensive disorder of pregnancy characterized by elevated blood pressure (BP) ≥140/90 mmHg in mild cases and ≥160/110 mmHg in severe cases. Vitamin D3 insufficiency or deficiency has been observed in patients diagnosed with preeclampsia, suggesting a potential link between low vitamin D levels and hypertensive disorders during pregnancy (61). One proposed mechanism for this association is vitamin D3’s regulatory role in the renin-angiotensin-aldosterone system (RAAS). Vitamin D inhibits renin production, preventing excessive RAAS activation, which otherwise leads to increased blood pressure, renal vasoconstriction, and sodium and water retention (61). Therefore, low vitamin D3 levels may contribute to RAAS overactivation, potentially increasing the risk of hypertensive complications in pregnancy. Despite these findings, the effectiveness of vitamin D3 supplementation in preventing preeclampsia remains controversial. While some systematic reviews suggest that vitamin D supplementation may reduce preeclampsia risk, other studies report inconclusive results.

A large-scale study involving 2,969 pregnant women demonstrated that vitamin D3 supplementation significantly reduced preeclampsia risk by nearly 50%, emphasizing its potential as a preventive maternal health measure (62). However, other studies have failed to establish a statistically significant protective effect, suggesting that additional factors, such as genetic predisposition, diet, sun exposure, and body mass index (BMI), may influence the outcomes of vitamin D supplementation in pregnancy (63).

Similarly, a cohort study examining the impact of vitamin D3 supplementation in _d_eficient pregnant women found that while supplementation restored normal vitamin D levels, no significant reduction in adverse perinatal events was observed (64). These conflicting results highlight the need for further large-scale, well-controlled clinical trials to determine whether vitamin D supplementation provides consistent benefits in reducing preeclampsia incidence.

### 3.7 Vitamin D importance in patients with chronic kidney disease (CKD)

#### 3.7.1 Vitamin D supplementation in hyperparathyroidism associated to CKD

In patients with chronic kidney disease (CKD), D3 deficiency has been identified as a risk factor for mortality. This is explained by the fact that the kidney plays a key role in the conversion of 25-hydroxyvitamin D to 1.25-dihydroxyvitamin D (calcitriol), which is essential for the activation of vitamin D receptors. This activation provides protection against the development of hyperparathyroidism, hypertension (HTN), renal damage, and systemic inflammation (65). In this regard, a prospective, double-blind, randomized, placebo-controlled trial conducted on 45 patients found that the group receiving true D3 supplementation with daily doses of 8,000 IU/day showed a reduction in parathyroid hormone (PTH) levels. It was concluded that in these cases, D3 supplementation acts as a protective factor against the development of hyperparathyroidism (66).

In a study conducted by Dogan et al., it was observed that 300,000 IU of colecalciferol per month orally caused a statistically significant increase in calcidiol levels (*p* < 0.001), as well as a decrease in iPTH levels. The results supported the proposed hypothesis, concluding that patients with CKD greatly benefit from D3 supplementation (67).

#### 3.7.2 Vitamin D supplementation in patients with CDK and diabetes mellitus

In a cohort study involving 344 patients with CKD, of whom 103 had diabetes and 240 did not, identified a protective effect of D3 supplementation. The findings showed a significant association between supplementation and reduced levels of HbA1C, suggesting its potential role in improving glycemic control in this population (68). The findings suggest that vitamin D3 may play a supportive role in managing glycemic levels, offering potential benefits for this population (69). This highlights the protective role of D3 in improving survival outcomes. Additionally, it has been suggested that daily D3 supplementation may help reduce albuminuria in CKD patients, a key marker of kidney damage. This reduction not only benefits renal health but also contributes to better overall outcomes for this population, reinforcing the significance of addressing D3 deficiency in their management (70).

### 3.8 Vitamin D and cystic fibrosis

It is estimated that around 98% of patients with cystic fibrosis (CF) experience vitamin D insufficiency, making it a significant concern in this population. A systematic review evaluated the effectiveness and safety of high-dose vitamin D supplementation in correcting inadequate serum 25-hydroxyvitamin D (25(OH)D) levels in adults with CF (71). The findings revealed that individuals receiving high-dose cholecalciferol achieved an average serum 25(OH)D concentration of 40.19 ng/mL ± 10.21 ng/mL, compared to 28.50 ng/mL ± 7.35 ng/mL in the control group (*p* < 0.01), demonstrating the efficacy of high-dose supplementation in reaching optimal 25(OH)D levels (>30 ng/mL) (71). Dosing regimens varied, ranging from daily supplementation of 1,700 IU to a single 250,000 IU dose. Importantly, no cases of hypervitaminosis D or hypercalcemia were reported, and adverse effects were minimal, supporting the safety and benefits of high-dose vitamin D supplementation in adults with CF (71).

Cholecalciferol, or vitamin D3, is naturally found in animal sources such as tuna, other fatty fish, eggs, and fortified foods including milk and cereals. Regarding supplementation, vitamin D3 has been found to be more effective than vitamin D₂ in achieving and maintaining serum 25(OH)D levels above 30 ng/mL. According to the European Cystic Fibrosis Society, infants should be started on vitamin D₂ or D3 at a dose of 1,000–2,000 IU per day, while children older than 1 year and adults are recommended to take 1,000–5,000 IU per day (72).

### 3.9 Role of vitamin D in the immune system

#### 3.9.1 Vitamin D and the innate and adaptive immune system

The nuclear VDR binds to vitamin D and regulates the expression of specific immune-related genes. VDR is expressed by various immune cells, including monocytes, macrophages, B and T lymphocytes, and dendritic cells. Additionally, the enzyme α-1-hydroxylase, responsible for converting vitamin D into its active form, is present in these cells, allowing for autocrine and paracrine immune modulation (73).

Through this mechanism, vitamin D downregulates pro-inflammatory cytokines such as TNF-α, IL-6, IL-8, MCP-1, and IL-12 while also reducing excessive reactive oxygen species by increasing intracellular glutathione levels (73).

Adaptive immune responses, initiated by antigen-presenting cells such as dendritic cells, B cells, and macrophages, are also influenced by vitamin D. The active form of vitamin D, 1,25(OH)₂D, has been shown to induce regulatory T cells (Tregs), which contribute to immune tolerance and help prevent cytokine storms associated with severe viral infections (73). Furthermore, vitamin D regulates adaptive immunity by limiting dendritic cell maturation and antigen presentation, shifting T cell differentiation from the pro-inflammatory Th1 and Th17 subsets toward Th2 and Tregs, which are involved in suppressing excessive immune responses (73).

#### 3.9.2 Vitamin D in antimicrobial regulation

Activation of pattern recognition receptors, such as Toll-like receptors (TLRs) and NOD-like receptors (NLRs), increases the expression of VDR and α-1-hydroxylase in immune cells. This activation enhances antimicrobial responses by controlling pro-inflammatory cytokine production, promoting autophagy, and increasing the synthesis of antimicrobial peptides like cathelicidin and β-defensins (73).

Cathelicidin LL-37, produced by neutrophils and epithelial cells, plays a key role in antimicrobial defense, neutralizing lipopolysaccharides (LPS) and contributing to wound healing, angiogenesis, and clearance of dead cells. Vitamin D stimulates LL-37 production, further supporting its role in immune defense. In keratinocytes and macrophages, TLR2 activation leads to increased conversion of vitamin D to its active form, enhancing microbicidal activity in both skin and circulating phagocytes (74).

Vitamin D metabolism is also influenced by cytochrome P450scc (CYP11A1), which converts vitamin D into the non-calcemic analogue 20S-hydroxyvitamin D3. This metabolite has potential therapeutic applications, including the treatment of rheumatoid arthritis and reducing inflammatory cytokines. CYP11A1 is expressed in immune cells, including CD4 and CD8 T lymphocytes, B lymphocytes, and monocytes, playing a role in local steroidogenesis and immune regulation, particularly in maintaining skin integrity and immune function (75).

#### 3.9.3 Vitamin D in defense against infections

Vitamin D plays a pivotal role in hosting immune response to infections, particularly by enhancing the innate immune system. Upon bacterial exposure, monocytes and macrophages upregulate the expression of VDR and the enzyme 1-alpha-hydroxylase, which locally converts 25-hydroxyvitamin D to its active form, calcitriol. This promotes the synthesis of antimicrobial peptides—such as defensins—that impair intracellular pathogen survival (76). Additionally, vitamin D supports autophagy, a key intracellular defense mechanism that isolates and degrades pathogens and damaged organelles through lysosomal processing. This process also enhances antigen presentation to T lymphocytes, thereby promoting immune surveillance and maintaining homeostasis (73).

Although these mechanistic insights underscore the immunological relevance of vitamin D, a direct causal relationship between serum vitamin D levels and infection susceptibility remains unconfirmed. While vitamin D modulates multiple aspects of the immune response, current evidence does not support its supplementation solely for infection prevention, given the inconsistent outcomes reported in clinical studies (76).

Vitamin D also contributes to antiviral defense by upregulating antimicrobial peptides such as cathelicidin (LL-37) and β-defensins, which disrupt viral envelopes and inhibit viral replication both *in vitro* and *in vivo* (77). In parallel, calcitriol modulates cytokine signaling pathways: it increases IκBα expression, which inhibits NF-κB activation and reduces proinflammatory cytokine production during infections such as respiratory syncytial virus (RSV). Moreover, vitamin D promotes the differentiation of monocytes into macrophages with enhanced chemotactic and phagocytic capabilities, facilitating pathogen clearance while limiting immunopathology (78).

Meta-analyses of randomized controlled trials have shown that daily or weekly vitamin D supplementation significantly reduces the incidence of acute respiratory tract infections, particularly in individuals with baseline vitamin D deficiency. However, bolus dosing strategies appear less effective in this context (78). Observational and interventional studies also report that low serum 25(OH)D concentrations are associated with increased severity of influenza, RSV, and COVID-19. Conversely, vitamin D supplementation has been linked to reduced intensive care unit (ICU) admissions and lower mortality rates among hospitalized COVID-19 patients (79, 80). These findings collectively suggest that maintaining sufficient vitamin D status may enhance first-line antiviral defenses and mitigate the severity of infectious diseases (81).

Emerging evidence further emphasizes the relevance of vitamin D in both acute COVID-19 outcomes and post-infection recovery. A 2020 study reported that critically ill COVID-19 patients with serum 25(OH)D levels below 12 ng/mL exhibited a 3.2-fold increased risk of mortality compared to patients with levels above 30 ng/mL (82). This supports the proposed immunomodulatory role of vitamin D in attenuating cytokine storms and preserving pulmonary epithelial integrity. More recently, a 2023 intervention trial demonstrated that high-dose cholecalciferol supplementation (50,000 IU/week for 8 weeks) significantly improved neuropsychiatric symptoms in post-COVID-19 patients. Compared to controls, those receiving supplementation experienced a 42% reduction in fatigue, 38% reduction in anxiety, and 35% improvement in cognitive dysfunction (83). These findings suggest a dual role for vitamin D—not only in modulating acute immune responses during infection but also in facilitating neurological recovery during long COVID (84).

#### 3.9.4 Vitamin D and its relationship with immune system diseases

The active form of vitamin D, 1,25-dihydroxyvitamin D, acts as an immune modulator by inhibiting dendritic cell maturation and reducing excessive activation of the acquired immune system. Its deficiency has been associated with an increased risk of autoimmune diseases, including psoriasis, type 1 diabetes, multiple sclerosis, and inflammatory bowel disease (76). Vitamin D supplementation is often incorporated into treatment strategies for these conditions (75).

The relationship between vitamin D status and allergic diseases, such as asthma and eczema, remains inconclusive. Some studies suggest vitamin D supplementation may reduce wheezing and improve forced expiratory volume in the first second (FEV1), but its efficacy in asthma prevention and treatment is still uncertain (76).

Routine screening for vitamin D deficiency is not recommended for the general population but is advised for individuals at higher risk, including those with hyperparathyroidism, hypoparathyroidism, osteoporosis, or kidney disease. Vitamin D deficiency is particularly common in older adults and has been associated with increased levels of proinflammatory cytokines, a higher incidence of pneumonia, and an elevated risk of upper respiratory tract infections (73).

### 3.10 Potential therapeutic uses of vitamin D

#### 3.10.1 Vitamin D and neurological health

Vitamin D plays an important role in the nervous system due to the presence of 1α-hydroxylase, 25-hydroxylase, and nuclear VDRs in key brain regions, including the thalamus, nucleus accumbens, stria terminalis, and amygdala (85). This enables local production of vitamin D, which regulates neurotrophin expression, neural differentiation, neurotransmission, synaptic plasticity, and apoptosis inhibition (86–88). Multiple studies have linked vitamin D deficiency to neurological diseases, including multiple sclerosis, Alzheimer’s disease (AD), Parkinson’s disease (PD), and amyotrophic lateral sclerosis, as well as cognitive decline, increased stroke risk, and carotid atherosclerosis (89, 90). Vitamin D deficiency has also correlated with cognitive decline, increased stroke risk, and carotid atherosclerosis (91). Nonetheless, while the association between vitamin D deficiency and neurological disorders is well-documented, the efficacy of vitamin D supplementation in treating or preventing these conditions remains unclear, necessitating further clinical trials (92).

In AD, vitamin D may play a role in pathogenesis by modulating immune response, oxidative stress, and mitochondrial function (90, 93). Observational studies have shown that active vitamin D metabolites enhance amyloid-β clearance by macrophages (94, 95). However, clinical trials have reported mixed results; one found no cognitive benefits from high-dose vitamin D supplementation in mild-to-moderate AD patients (96), while others suggest maintaining sufficient vitamin D levels may help prevent or slow cognitive decline (97, 98). More well-designed randomized controlled trials (RCTs) are needed to confirm vitamin D’s role in AD treatment and prevention.

In Parkinson’s disease, low serum 25(OH)D₃ levels have been inversely associated with disease severity and motor symptom progression (99, 100), as measured by the Unified Parkinson’s Disease Rating Scale (UPDRS) and Hoehn and Yahr Scale (101–103). Although a causal link has not been established, preclinical studies suggest that vitamin D may exert neuroprotective effects on dopaminergic neurons in the substantia nigra by reducing oxidative stress, inhibiting neuroinflammation, and acting as a neurotrophic factor (104).

Clinical trials report inconsistent results. Some studies demonstrate symptomatic improvement with vitamin D supplementation (99, 105–108), while others find no significant benefit (109, 110). Genetic variability may explain these discrepancies; for example, a 12-month double-blind placebo-controlled trial involving 104 PD patients showed that those with the *FokI* TT allele (rs10735810) experienced greater motor improvement after receiving 1,200 IU/day of vitamin D, highlighting the influence of VDR polymorphisms on treatment response (105). These findings suggest that vitamin D may offer therapeutic benefits in PD management, particularly in genetically susceptible individuals, although further validation is required.

Recent research by Li et al. also indicates that vitamin D may exert epigenetic effects in the brain. For instance, DNA methylation changes in AD-related genes such as *BACE1* may reduce Aβ production, contributing to neuroprotection (111). In parallel, vitamin D supports mitochondrial integrity by stabilizing mitochondrial membrane potential and decreasing ROS—mechanisms relevant to the pathogenesis of neurodegenerative diseases. Clinically, higher serum vitamin D levels have been associated with slower cognitive decline, potentially through upregulation of neurotrophic factors such as brain-derived neurotrophic factor (BDNF) and enhancement of acetylcholine synthesis (112). However, intervention trials remain inconsistent. A study administering 4,000 IU/day of vitamin D to patients with mild-to-moderate AD failed to demonstrate cognitive improvement, possibly due to the advanced stage of disease or irreversible neuronal damage (113). In contrast, subgroup analyses suggest that individuals with baseline vitamin D deficiency may derive greater benefit, reinforcing the importance of early intervention and individualized dosing strategies.

#### 3.10.2 Vitamin D in dermatological disorders

Vitamin D is synthesized in basal skin cells when exposed to UV light and has antiproliferative, pro-differentiating, immunomodulatory, and anti-apoptotic effects on keratinocytes, helping to prevent opportunistic infections (114). In fact, an inverse correlation has been observed between serum vitamin D levels and *Staphylococcus aureus* skin colonization after 4 weeks of supplementation (115). Low vitamin D levels are also linked to autoimmune skin diseases, increased severity of atopic dermatitis, and chronic urticaria (116, 117). Emerging evidence suggests vitamin D may help prevent skin malignancies and have therapeutic applications in atopic dermatitis, psoriasis, and related skin disorders (116, 118), though further research is needed.

In psoriasis, lower 1,25-dihydroxyvitamin D₃ levels have been reported compared to healthy controls (119–121). Vitamin D may influence psoriasis pathogenesis by regulating keratinocyte proliferation and maturation (122). Topical vitamin D has become an important therapeutic option, with VDR mRNA expression in psoriatic lesions correlating with its antiproliferative activity (123, 124). However, the benefits of oral vitamin D supplementation remain unclear (125). While some studies reported improvements in Psoriasis Area and Severity Index (PASI) scores, recent meta-analyses, including Formisano et al., found no significant differences, highlighting the need for large-scale clinical trials to determine optimal dosing and therapeutic efficacy (119).

Research on vitamin D supplementation in atopic dermatitis (AD) has yielded mixed results. By downregulating pro-inflammatory cytokines (IL-6, TNF-α) and enhancing antimicrobial peptide production (cathelicidin), vitamin D may improve skin barrier function and immune regulation, reducing infection susceptibility in AD patients (77). Some trials found significant improvements in AD severity, as measured by Eczema Area and Severity Index (EASI) and Scoring Atopic Dermatitis (SCORAD), following cholecalciferol (vitamin D3) supplementation (126–128). It has been particularly beneficial for winter-related AD in children (129). A RCT by El-Heis et al. provided evidence that antenatal cholecalciferol supplementation reduced the risk of infantile eczema (130). However, not all studies found supplementation effective, as vitamin D levels correlated with AD severity but did not consistently improve symptoms (131, 132).

#### 3.10.3 Vitamin D in pulmonary diseases

Vitamin D plays a critical role in the respiratory system, with evidence from animal models showing that 1,25-dihydroxyvitamin D enhances alveolar fluid clearance and reduces pulmonary edema by decreasing vascular permeability and regulating sodium channel (ENaC) expression (133). Additionally, its immunomodulatory and anti-inflammatory properties, including attenuation of LPS signaling, may influence lung fibroproliferation and remodeling (134). Vitamin D also affects innate and adaptive immunity, promoting the production of antimicrobial peptides such as cathelicidin (LL-37) and β-defensins (135–137). These findings suggest a potential role for vitamin D in preventing and managing respiratory infections, chronic obstructive pulmonary disease (COPD), asthma, and lung cancer (138).

In respiratory infections, vitamin D enhances antibacterial activity in monocytes. Locally synthesized 1,25(OH)₂D binds to VDR in immune cells, regulating antimicrobial peptide production (LL-37, β-defensins), pattern recognition receptors (PRRs), Toll-like receptors, and pro-inflammatory cytokines (IL-6, TNF-α) (81). Vitamin D also induces autophagy in immune cells, potentially aiding in the control of intracellular pathogens like *M. tuberculosis* (139). Epidemiological studies associate low vitamin D levels with a higher risk of respiratory infections, including COVID-19 (1.77 times greater risk in deficient individuals) (140) and tuberculosis (141, 142). However, randomized controlled trials on vitamin D supplementation for respiratory infections have shown mixed results, with both positive effects (143–145) and no significant benefit (129, 146–151).

The role of vitamin D in airway remodeling asthma and COPD is an active area of research. A systematic review by Salameh et al. highlighted vitamin D’s immunomodulatory effects in asthma pathogenesis, particularly its inhibition of matrix metalloproteinases (MMP-9, ADAM33), collagen synthesis, smooth muscle contractions, and nuclear factor kappa B (NF-κB), all of which are involved in airway remodeling (152). Additionally, vitamin D’s regulation of oxidative stress, protease/antiprotease balance, and tissue repair mechanisms may play a role in chronic lung disease progression (153). Given its broad biological activity, vitamin D supplementation may offer therapeutic benefits in asthma and COPD, though well-designed interventional studies are needed to confirm its clinical efficacy (154).

#### 3.10.4 Vitamin D in Cancer prevention and treatment

Epidemiological studies have consistently linked low circulating levels of 25-hydroxyvitamin D [25(OH)D] with an increased risk of various malignancies, including cancers of the oral cavity, breast, prostate, endometrium, ovary, and colorectum (155). The biologically active form of vitamin D, calcitriol (1,25-dihydroxyvitamin D₃), exhibits potent anticancer properties in preclinical studies. These include antiproliferative, pro-apoptotic, anti-angiogenic, and anti-invasive effects, largely mediated through activation of the VDR. VDR signaling regulates the expression of genes involved in cell cycle arrest, cellular differentiation, apoptosis, epithelial-mesenchymal transition (EMT), and the maintenance of cancer stem cells (156, 157). Experimental studies demonstrate that calcitriol and its analogs can synergize with standard chemotherapeutic agents. For instance, co-administration of tacalcitol (PRI-2191) with 5-fluorouracil significantly suppresses colon tumor growth in preclinical models, outperforming either agent used alone (158), and calcitriol enhances cytarabine-induced DNA fragmentation and leukemic cell death in acute myeloid leukemia model (159).

Despite the compelling preclinical evidence, large-scale randomized, placebo-controlled trials evaluating the efficacy of daily vitamin D₃ supplementation in cancer prevention and treatment have yielded mixed results. Most notably, these trials have not demonstrated a statistically significant reduction in overall cancer incidence, and only a modest (approximately 6%), non-significant reduction in cancer-related mortality has been reported in pooled analyses (160). However, subgroup analyses from these trials suggest that consistent daily dosing—as opposed to intermittent high-dose bolus administration—may be more effective, with estimates indicating up to a 12% reduction in cancer-specific mortality (161).

Calcitriol’s ability to enhance the therapeutic efficacy of cytotoxic agents has also been confirmed in multiple laboratory settings. In colon cancer models, it improves the response to 5-fluorouracil (162), and in acute myeloid leukemia, it enhances the apoptotic activity of cytarabine (163). However, systematic reviews and meta-analyses of randomized, placebo-controlled trials found that daily vitamin D supplementation did not significantly impact cancer mortality compared to controls (161).

Laboratory studies have consistently demonstrated that vitamin D, particularly its active form calcitriol (1,25-dihydroxyvitamin D), exhibits anticancer properties through various mechanisms. These include promoting cell cycle arrest, inducing apoptosis, inhibiting angiogenesis, and modulating immune responses, all of which contribute to the suppression of tumor growth and metastasis in preclinical models (164). Despite these promising findings, large-scale randomized controlled trials (RCTs) have not consistently shown a significant reduction in overall cancer mortality with vitamin D supplementation (161).

Several factors may account for this discrepancy. Many RCTs have been conducted in populations with sufficient baseline vitamin D levels, potentially limiting the observable benefits of supplementation. Additionally, variations in dosing regimens, such as daily versus intermittent bolus dosing, may influence outcomes; some analyses suggest that daily dosing is more effective in reducing cancer mortality (161).

#### 3.10.5 Vitamin D and psychiatric disorders

Systematic reviews of observational studies and randomized controlled trials report that hypovitaminosis D significantly correlated with schizophrenia (165), depression (166, 167), autism spectrum disorder (168), delirium in hospitalized patients and other psychiatric disorders (169). Vitamin D plays crucial roles in brain development, neurotransmitter regulation, and neuroprotection (170). In fact, observational evidence demonstrates lower serum 25-hydroxyvitamin D (25OHD) is related to poorer cognition and predict executive dysfunctions (mental shifting, information updating and processing speed) (171). Vitamin D receptors in the brain influence critical pathways involved in mood regulation, including those affecting neurotransmitters like serotonin and dopamine (88).

Mechanistically, vitamin D regulates serotonin synthesis by modulating the expression of tryptophan hydroxylase 2 (TPH2), a key enzyme in serotonin production (172). Indeed, a systematic review of randomized controlled trials in which cholecalciferol supplementation ranged from 600 to 300,000 IU daily for 6 weeks to 2 years found a significant reduction in depressive symptoms, assessed by the Beck Depression Inventory (BDI), Geriatric Depression Scale (GDS), Hamilton Depression Rating Scale (HDRS), or Montgomery-Åsberg Depression Rating Scale (MADRS), particularly in individuals with severe baseline deficiency (<50 nmol/L) (173).

### 3.11 Public health strategies, screening recommendations, and global health relevance

Despite the well-established physiological functions of vitamin D, public health strategies to prevent and manage its deficiency remain underdeveloped in many regions worldwide. Vitamin D deficiency is highly prevalent on a global scale. Approximately 15.7% of the global population has serum 25-hydroxyvitamin D [25(OH)D] concentrations below 30 nmol/L (12 ng/mL), and nearly 50% have levels below 50 nmol/L (20 ng/mL), indicating widespread insufficiency and underscoring the need for effective public health interventions (174).

Food fortification has emerged as a cost-effective and scalable strategy to improve vitamin D status at the population level. Fortifying commonly consumed staple foods—such as milk, bread, and eggs—has been shown to significantly increase serum 25(OH)D levels across diverse demographic groups. Advances in encapsulation and stabilization technologies have enhanced the bioavailability and shelf-stability of vitamin D in fortified products, making this approach more efficient and sustainable than pharmaceutical supplementation in many settings. Countries that have implemented mandatory or voluntary fortification policies have reported substantial improvements in population-wide vitamin D status (175).

In terms of screening practices, current guidelines do not support universal screening for vitamin D deficiency in asymptomatic individuals, due to a lack of conclusive evidence demonstrating improved health outcomes from such an approach. Instead, targeted screening is recommended for high-risk populations. These include individuals with limited sun exposure, darker skin pigmentation, malabsorptive conditions (e.g., celiac disease, inflammatory bowel disease), chronic kidney or liver disease, and those living at higher latitudes where sunlight exposure is minimal for extended periods (19). Screening in these groups allows for early identification and personalized interventions to prevent complications related to severe deficiency.

## 4 Conclusion

Vitamin D₃ (cholecalciferol) exerts broad physiological effects that extend well beyond its classical role in calcium-phosphorus homeostasis and skeletal health. Evidence from epidemiological, mechanistic, and clinical studies suggests that adequate vitamin D status is associated with favorable outcomes across a wide range of systems, including cardiovascular, immune, gastrointestinal, metabolic, reproductive, and neurological domains. Deficiency in serum 25-hydroxyvitamin D has been linked to increased risks of cardiovascular disease, inflammatory bowel disease, insulin resistance, fractures, neurodegenerative disorders such as Alzheimer’s and Parkinson’s, pregnancy complications, and certain cancers.

Recent findings highlight vitamin D’s role in modulating immune responses, enhancing gut barrier integrity, regulating glucose metabolism, and potentially attenuating neuroinflammation and tumor progression. However, despite promising preclinical data, the results of large-scale randomized controlled trials remain inconclusive, particularly regarding its impact on cancer incidence and mortality, cognitive decline, and long-term metabolic outcomes.

These inconsistencies underscore the need for a cautious and evidence-based approach to vitamin D supplementation. While food fortification and targeted supplementation have proven effective in improving population-level vitamin D status, universal high-dose supplementation is not warranted and may pose risks, including hypercalcemia and nephrolithiasis. Personalized strategies—guided by serum 25(OH)D measurements and individual risk factors such as age, comorbidities, body mass index, and absorption capacity—are essential for optimizing therapeutic benefit while minimizing harm.

Vitamin D represents a promising yet complex component of preventive and therapeutic strategies in human health. Further well-designed, large-scale clinical trials are needed to clarify its efficacy in disease prevention and management, define optimal dosing regimens, and guide public health policies tailored to population needs.
