# Supplementary material for: Cholecalciferol (vitamin D3): efficacy, safety, and implications in public health
Source: Front Nutr. 2025 Jun 9;12:1579957. doi: 10.3389/fnut.2025.1579957 (PMC12183072; doi:10.3389/fnut.2025.1579957)
Supplement: Supplementary file 1 [file Table_1.docx]

**Table S1:** CASP Checklist: For Randomised Controlled Trials

| **Study** | **1** | **2** | **3** | **4** | **5** | **6** | **7** | **8** | **9** | **10** | **11** | **12** | **13** |
| --- | --- | --- | --- | --- | --- | --- | --- | --- | --- | --- | --- | --- | --- |
| Lauresne et al. (18) | YES | YES | YES | NO | CANT TELL | YES | YES | YES | YES | YES | CANT TELL | YES | YES |
| Neale et al. (34). | YES | YES | YES | YES | YES | YES | YES | YES | YES | YES | YES | YES | YES |
| Mathur et al. (48). | YES | YES | YES | YES | YES | YES | YES | YES | YES | YES | YES | YES | YES |
| Rasouli et al. (49). | YES | YES | YES | YES | YES | YES | YES | YES | YES | YES | YES | YES | YES |
| Median et al. (71). | YES | YES | YES | YES | YES | YES | YES | YES | YES | YES | YES | YES | YES |
| Charoenporn (88) | YES | YES | YES | YES | YES | YES | YES | YES | YES | YES | YES | YES | YES |

1. Did the study address a clearly formulated research question?
2. Was the assignment of participants to interventions randomised?
3. Were all participants who entered the study accounted for at its conclusion?
4. Were the participants ‘blind’ to intervention they were given?
5. Were the investigators ‘blind’ to the intervention they were giving to participants?
6. Were the people assessing/analysing outcome/s ‘blinded’?
7. Were the study groups similar at the start of the randomised controlled trial?
8. Apart from the experimental intervention, did each study group receive the same level of care (that is, were they treated equally)?
9. Were the effects of intervention reported comprehensively?
10. Was the precision of the estimate of the intervention or treatment effect reported?
11. Do the benefits of the experimental intervention outweigh the harms and costs?
12. Can the results be applied to your local population/in your context?
13. Would the experimental intervention provide greater value to the people in your care than any of the existing interventions?

**Table S2:** NEWCASTLE - OTTAWA QUALITY ASSESSMENT SCALE

| **Study** | **1** | **2** | **3** | **4** | **5** | **6** | **7** | **8** | **Score** |
| --- | --- | --- | --- | --- | --- | --- | --- | --- | --- |
| Abraham et al. (42) | * | * | * | * | ** | * | * | * | 9 |
| Kiet et al. (44) | * | * | * | * | ** | * | * | * | 9 |
| Hyppönen et al. (67) | * | * | * | * | ** | * | * | * | 9 |
| Fernández et al (68) | * | * | * | * | * | * | * | * | 8 |
| Farías-Jofré(69) | * | * | * | * | * | * | * | * | 8 |
| Blair et al (73) | * | * | * | * | ** | * | * | * | 9 |

**Note:** A study can receive a maximum of one star for each item numbered within the Selection and Result categories. A maximum of two stars can be awarded for comparability.

**Selection**

1. Representativeness of the exposed court.

2. Selection of the unexposed court.

3. Exposure determination.

4. Demonstration that the current outcome of interest was not present at baseline.

**Comparability**

5. Cohort comparability based on design or analysis.

**Results**

6. Evaluation of the result.

7. Was the follow-up long enough for the results to occur?

8. Adequacy of cohort follow-up.

**Interpretation**

Good quality: 3 or 4 stars in the selection domain and 1 or 2 stars in the comparability domain and 2 or 3 stars in the outcome/exposure domain.

Acceptable quality: 2 stars in the selection domain and 1 or 2 stars in the comparability domain and 2 or 3 stars in the outcome/exposure domain.

Poor quality: 0 or 1 star in the selection domain or 0 stars in the comparability

domain or 0 or 1 stars in the outcome/ exposure domain

**Table S3:** NEWCASTLE - OTTAWA QUALITY ASSESSMENT SCALE

| **Study** | **1** | **2** | **3** | **4** | **5** | **6** | **7** | **8** | **9** | **10** | **Score** |
| --- | --- | --- | --- | --- | --- | --- | --- | --- | --- | --- | --- |
| Dogan et al. (72). | * |  | * | * | * | * | * | * | * | * | 9 |

**Is the Case Definition Adequate?**

1. Requires some independent validation (e.g. >1 person/record/time/process to

extract information, or reference to primary record source such as x-rays or

medical/hospital records)

2. Record linkage (e.g. ICD codes in database) or self-report with no reference to

primary record

3. No description

**Representativeness of the Cases**

4. All eligible cases with outcome of interest over a defined period of time, all cases

in a defined catchment area, all cases in a defined hospital or clinic, group of

hospitals, health maintenance organization, or an appropriate sample of those

cases (e.g. random sample)

5. Not satisfying requirements in part (a), or not stated.

**Selection of Controls**

6. Community controls (i.e. same community as cases and would be cases if had

outcome)

7. Hospital controls, within same community as cases (i.e. not another city) but

derived from a hospitalized population

8. No description

**4 Definition of Controls**

9. If cases are first occurrence of outcome, then it must explicitly state that controls

have no history of this outcome. If cases have new (not necessarily first)

occurrence of outcome, then controls with previous occurrences of outcome of

interest should not be excluded.

10. No mention of history of outcome
